# Fermi Level shifting, Charge Transfer and Induced Magnetic Coupling at La_0.7_Ca_0.3_MnO_3_/LaNiO_3_ Interface

**DOI:** 10.1038/srep08460

**Published:** 2015-02-13

**Authors:** Xingkun Ning, Zhanjie Wang, Zhidong Zhang

**Affiliations:** 1 Shenyang National Laboratory for Materials Science, Institute of Metal Research (IMR), Chinese Academy of Sciences (CAS), 72 Wenhua Road, Shenyang 110016, China

## Abstract

A large magnetic coupling has been observed at the La_0.7_Ca_0.3_MnO_3_/LaNiO_3_ (LCMO/LNO) interface. The x-ray photoelectron spectroscopy (XPS) study results show that Fermi level continuously shifted across the LCMO/LNO interface in the interface region. In addition, the charge transfer between Mn and Ni ions of the type Mn^3+^ − Ni^3+^ → Mn^4+^ − Ni^2+^ with the oxygen vacancies are observed in the interface region. The intrinsic interfacial charge transfer can give rise to itinerant electrons, which results in a “shoulder feature” observed at the low binding energy in the Mn 2*p* core level spectra. Meanwhile, the orbital reconstruction can be mapped according to the Fermi level position and the charge transfer mode. It can be considered that the ferromagnetic interaction between Ni^2+^ and Mn^4+^ gives rise to magnetic regions that pin the ferromagnetic LCMO and cause magnetic coupling at the LCMO/LNO interface.

Due to the coupling of the spin, charge and orbital freedom of 3*d* electrons, the research area that focuses on the interfaces between dissimilar complex oxide materials is attracting considerable attention^1^,^2^,^3^,^4^. These interfaces exhibit much richer physical connotation than conventional semiconductor heterostructures because of the novel electronic reconstruction and magnetic states. Recently, remarkable improvement in techniques for growing and characterizing oxide thin films has opened an avenue for the study of the new interfacial electronic states at the interface between ABO_3_ perovskite oxides. In these systems, novel physical properties such as 2-dimension (2D) superconductivities, artificial topological insulators and unexpected magnetic interaction have been found and envisaged as the promising ideal system for the realization of nanoscale oxide devices^5^,^6^,^7^,^8^,^9^.

The factors like orbital reconstruction of the 3*d* electrons and oxygen vacancies at the interface could drastically tune the interfacial properties^10^,^11^. Many researches are focused on the interface between the LaAlO_3_ and SrTiO_3_, in which researchers emphasized the importance of the oxygen vacancies to interpret the high density of the electron gas at the interface^10^,^12^,^13^,^14^. Salluzzo et al. indicated that the generation of conducting electron gas is related to an orbital reconstruction of the 3*d* electrons^15^. The charge transfer as another important factor is also discussed for the origin of the novel physical properties. In particular, recently, a considerable number of experiments have been carried out on the magnetic coupling effect in antiferromagnetic/Pauli paramagnetic (AFM/PM), ferromagnetic/antiferromagnetic (FM/AFM) and ferromagnetic/itinerant ferromagnetic (FM/FM) interfaces, such as CaMnO_3_/LaNiO_3_ (CMO/LNO), LaMnO_3_/LaNiO_3_ (LMO/LNO), La_0.7_Sr_0.3_MnO_3_/BiFeO_3_ (LSMO/BFO) and La_0.7_Sr_0.3_MnO_3_/SrRuO_3_ (LSMO/SRO), highlighting the charge transfer mechanism which causes the magnetic interaction at interfaces^16^,^17^,^18^,^19^. However, whether the charge transfer would lead to magnetic coupling in these systems is still a controversial issue. For example, in La_0.3_Sr_0.7_FeO_3_/SrRuO_3_ (LSFO/SRO) bilayers and LSMO/SRO superlattice, the charge transfer did not play a dominant role in determining the interfacial magnetic coupling^20^,^21^. In order to understand and explain the role of these factors for the unusual interfacial properties, the detailed spectroscopic studies on these interfaces will be needed.

The charge state in these strongly correlated electron oxides is closely coupled with the spin and orbital degrees of freedom. In addition, the charge transfer and orbital reconstruction will associate with charge neutrality and oxygen stoichiometry in the perovskite manganites^22^. Therefore, it is important to investigate the valence states of the 3*d*-ions at the interface to explore the relationship between the charge transfer and orbital reconstruction which could be used to explain the magnetic properties because the *e_g_*-electron transfer could effectively modulate the magnetic properties^23^,^24^. Recently, the charge transfer has been investigated by XPS at the LSMO/LNO, LMO/LNO and LSMO/YBCO interfaces^25^,^26^,^27^. However, on the one hand, the evolution of 3*d*-ions valence states in the interface region has not been illustrated. Exploring the continuous change of the valence states across the interface in the interface region can master a comprehensive picture of the charge transfer such as charge-transfer mode and scale^28^,^29^. On the other hand, the Fermi level position at the interface also should be investigated. In most cases, the interfacial nature such as charge transfer, orbital reconstruction *etc.* is determined by the prerequisite of the unifying of the Fermi level at interfaces^28^,^29^,^30^. Therefore, to investigate the Fermi level position at the interface can describe the charge transfer and orbital reconstruction, and further resolve the induced magnetic properties at the interface.

In this study, we select La_0.67_Ca_0.33_MnO_3_ (LCMO) films as the FM layer to investigate the charge transfer effect at the LCMO/LNO interface through the interfacial FM coupling. In this system, charge transfer and orbital reconstruction between the Mn and Ni ions will induce interfacial FM coupling. In addition to this, due to the metallic and insulating properties of the LNO and LCMO films at room temperature, a large build-in electric field will be formed in the LCMO/LNO interface region and the Fermi level will be unified at the interface due to the prerequisite of the energy continuity^31^,^32^. The evolutions of the Mn and Ni core level spectra and the Fermi level shifting in the interface region have been investigated by XPS. The experimental results indicate that the charge disproportionation as Mn^3+^ + Ni^3+^ → Mn^4+^ + Ni^2+^ combined with the O^2−^ ions transfer happened at the interface, and the Fermi level continuously shifts from LCMO to LNO across the interface. The map of the orbital structure shows that the Ni^2+^ − Mn^4+^ interaction is the key point to interpret the interfacial FM interaction in the LCMO/LNO bilayers.

## Results

**Figure 1:**
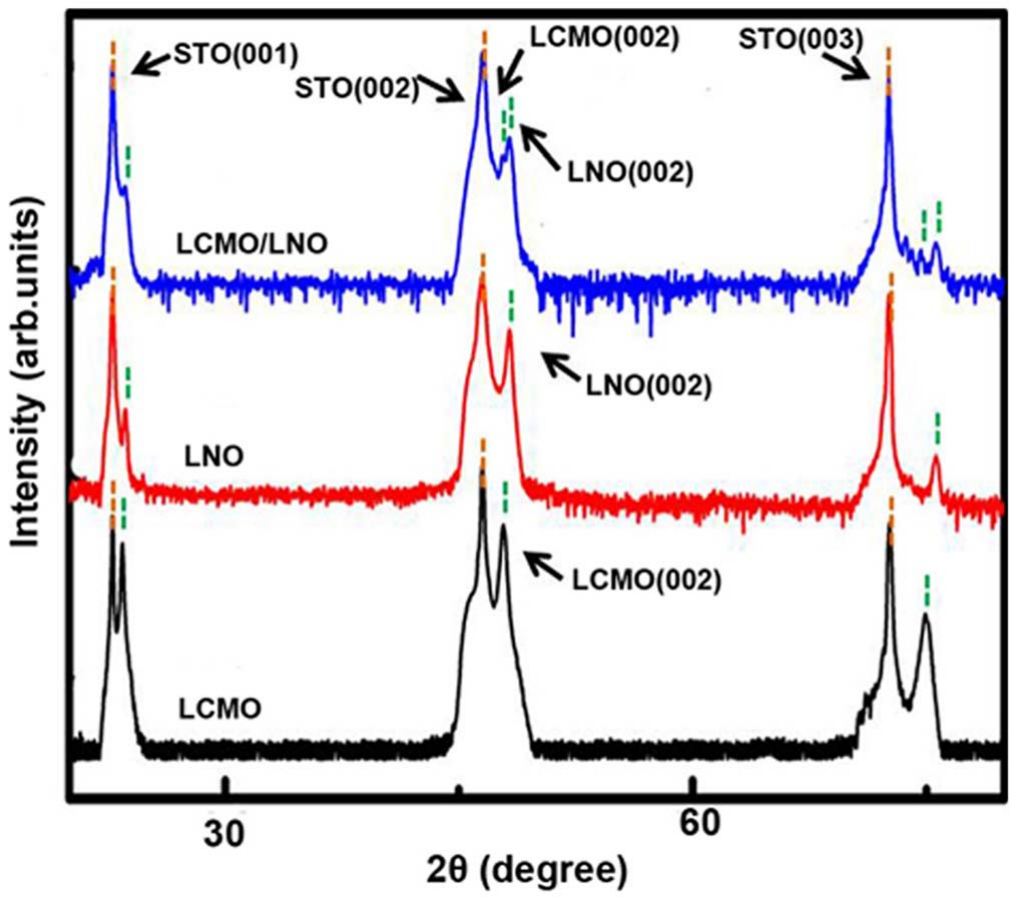
XRD patterns of the LCMO and LNO layers, and the LCMO/LNO bilayer.

Figure 1 shows the XRD patterns for the pure LCMO and LNO layers, and LCMO/LNO bilayer on the STO(001) substrates. All the layers are the perovskite phase and exhibit a (001)-preferred orientation, indicating that the LNO layer was epitaxially grown on the STO(001) substrate, and then the LCMO layer was epitaxially grown on the LNO layer. According to XRD analysis, the c-axis lattice constant of the LCMO and LNO layers is calculated as 3.850 and 3.830 Å, respectively, which are slightly smaller than the bulk values (3.857 Å for the LCMO and 3.850 Å for the LNO). The results indicate that both LCMO and LNO layers are under an in-plane tensile strain state. The lattice constant of the LCMO and LNO in the LCMO/LNO bilayers is a slight change, showing a smaller lattice mismatch between the LNO and LCMO layers at the interface. The transmission electron microscopy (TEM) study show that the thickness of the film is about 30 and 25 nm for LNO and LCMO layers, respectively. The interfaces of LNO/STO and LCMO/LNO are clear and flat. High-resolution TEM (HRTEM) images (Supplementary Fig. S1) also reveal the well-defined LNO/STO and LCMO/LNO interfaces, and in which it can be seen that both the LCMO and LNO layers have the same crystallographic orientation with respect to the STO substrate. The *d*-spacing of the out-of plane is determined to be 3.83 Å and 3.85 Å for LNO and LCMO, respectively, and consistent with the XRD results. It is noted that the relative strain is 0.5% for the LNO layer, and 0.2% for the LCMO layer calculated by comparing with the lattice constant of the bulks. The sharp interface between LCMO and LNO layers means that the interdiffusion was unlikely happened at the interface.

**Figure 2:**
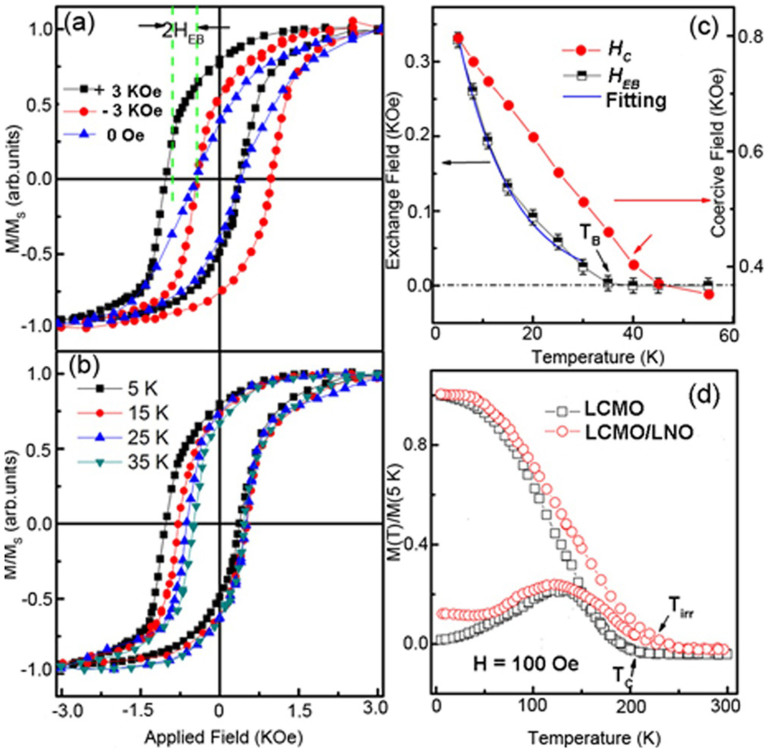
(a) Magnetic hysteresis loops of the LCMO/LNO bilayer at 5 K after ZFC and FC. Magnetization is normalized by the saturated value. (b) Magnetic hysteresis loops of the LCMO/LNO bilayer measured at different temperatures from 5 K to 35 K. (c) Exchange-bias field (*H_EB_*) and coercivity (*H_C_*) of the LCMO/LNO bilayer as a function of temperature. The exponential fitting of the exchange bias field (*H_EB_*) as a function of temperature is noted by the blue line. (d) Temperature dependence of magnetization of the LCMO/LNO bilayer and pure LCMO layer under an in-plane magnetic field of 100 Oe.

Magnetic properties of the LCMO/LNO bilayers were obtained by measuring the hysteresis loops at 5 K after field cooling (FC) from room temperature at a magnetic field of ±3 kOe. As shown in figure 2(a), it is clearly seen that the hysteresis loops shift along the magnetic-field axis, indicating that the exchange bias (EB) effect exists in the LCMO/LNO bilayer. The absolute values of the EB field (*H_EB_*) and coercivity (*H_C_*) are calculated using *H_EB_* = |*H_1_* + *H_2_*|/2 and *H_C_* = |*H_1_* − *H_2_*|/2, where *H_1_* and *H_2_* are the values of magnetic field at which the magnetization goes to zero. A large *H_EB_* of about 300 Oe is observed in the LCMO/LNO bilayer. The *H_EB_* of these samples after ZFC from room temperature is zero within the 10-Oe measurement resolution. The shift of the hysteresis loops is found to be highly reversible with respect to the field direction during FC, i.e., *H_EB_* ~ −300 Oe and *H_EB_* ~ 280 Oe is corresponds to the magnetic field of +3 kOe and −3 kOe, respectively. The ZFC magnetization loop is narrower than the FC loops. This is in line with the conventional EB observed in FM/AFM structures. In addition, the hysteresis loops of the LCMO/LNO bilayer measured at different temperatures show that both *H_EB_* and *H_C_* decrease with increasing temperature, as shown in figure 2(b). All the hysteresis loops at each temperature were measured after FC from room temperature. Figure 2(c) shows the variation of the *H_EB_* and *H_C_* with temperature from 5 K to 55 K. The *H_EB_* decreases almost monotonously with increasing temperature and become zero at about 35 K, which corresponds to conventional EB-blocking temperature (*T_B_*). The relation between *H_EB_* and temperature can be described by the following formula: *H_EB_* = *H*_0_ exp(−*T*/*T*_0_), where *H*_0_ is the extrapolation of *H_EB_* at 0 K and *T*_0_ is a constant. As shown in figure 2(c), *H_EB_* exponentially decay with increasing temperature. Similarly, *H_C_* decreases lineally with increasing temperature (figure 2(c)). It is noted that *H_C_* in particular shows a crossover at *T_B_*. This is in agreement with the experimental results in perovskite manganite in recent reports such as FM/AFM of La_1−x_Ca_x_MnO_3_/La_1−y_Ca_y_MnO_3_ superlattices^33^, FM/PM of LSMO/LNO bilayers^25^, FM/AFM of LSMO/SrMnO_3_^34^ and LSMO/NiO composite films^35^,^36^. This kind of thermal activation of the FM interactions across the interfaces has been proposed to manipulate the EB effect in the manganite systems. It have been reported by Cai *et al*.^37^ that the random spin coupling can be established in the PM states even with the films. The observed exchange bias and enhancement of coercivity in the bilayer unambiguously mean the existence of interfacial magnetic coupling in the LCMO/LNO interface even when the LNO is in the paramagnetic (PM) state. Figure 2(d) presents the temperature dependences of magnetization with the ZFC and FC procedure for both pure LCMO layer and LCMO/LNO bilayer with an extra in-plane magnetic field of 100 Oe. For both systems, magnetization decreases with increasing temperature, and a FM-to-PM transition is observed. The Curie temperature (*T_C_*) is determined to be 210 K for the pure LCMO layer, which is in line with the reported value of the LCMO films grown by PLD with the same thickness^38^. However, a small quantity of the magnetization value has been found at the PM temperature regions in the LCMO/LNO bilayer. In addition, the irreversibility temperature (*T_irr_*) is obviously above *T*_C_ of the pure LCMO layer. It is, indeed, found experimentally that the high-temperature local magnetic region formed at the PM state of the LCMO. This may due to the Mn-O-Ni FM coupling and will be discussed below in detail.

**Figure 3:**
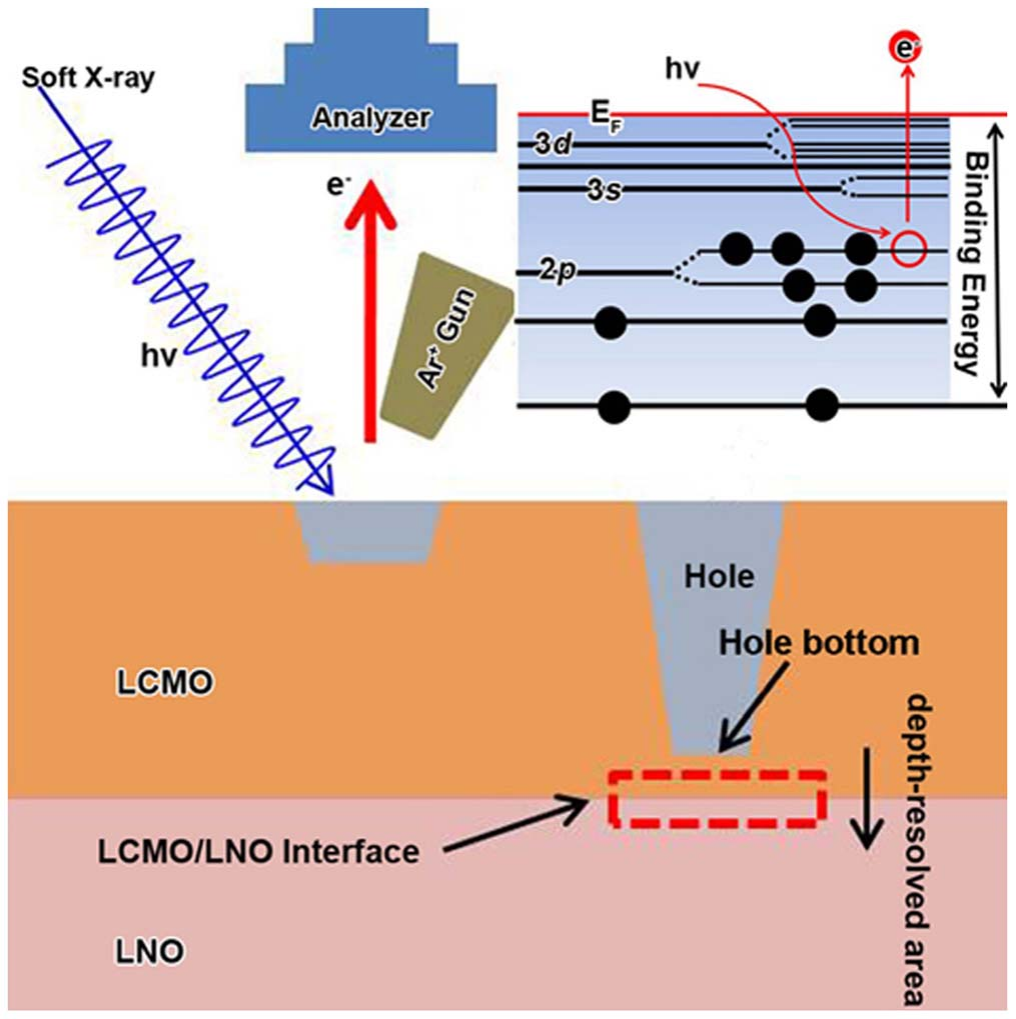
Schematic illustrations of the XPS procedures and the binding energies of different core-level electrons.

To reveal the origin of EB effect in the present LCMO/LNO bilayers, the evolutions of 3*d*-ions valence states and core-level spectra across the interface in the interface region have been studied by XPS. Thus, we can get the details of the charge distribution, and therefore discuss charge-transfer mode and range in the interface region. The Fermi level position at the interface can also be investigated. We utilized the following procedure, as shown in figure 3. Firstly, we investigated the core-level spectra of Mn ion of the LCMO layer from the surface to a depth of 10 nm using an Ar ion sputtering beam at 500 eV and found that they are keep nearly unchanged with increasing probing depth. Secondly, we dug a “hole” at the LCMO layer until near the interface about 4 nm using an Ar ion sputtering beam at 3000 eV. Then we investigated the core-level spectra of Mn ion at the hole bottom using the Ar ion sputtering beam at 500 eV, and compared them with the spectra obtained from the surface. We confirmed that the spectra characteristics at the hole bottom are the same as those obtained from the surface. Thirdly, the core-level spectra of Mn and Ni were investigated from the hole bottom to the LNO layer across the LCMO/LNO interface in the interface region (marked as the red box in figure 3). The XPS spectra were measured after etching time from 0 s to 300 s in the interface region using the Ar ion sputtering beam at 500 eV.

**Figure 4:**
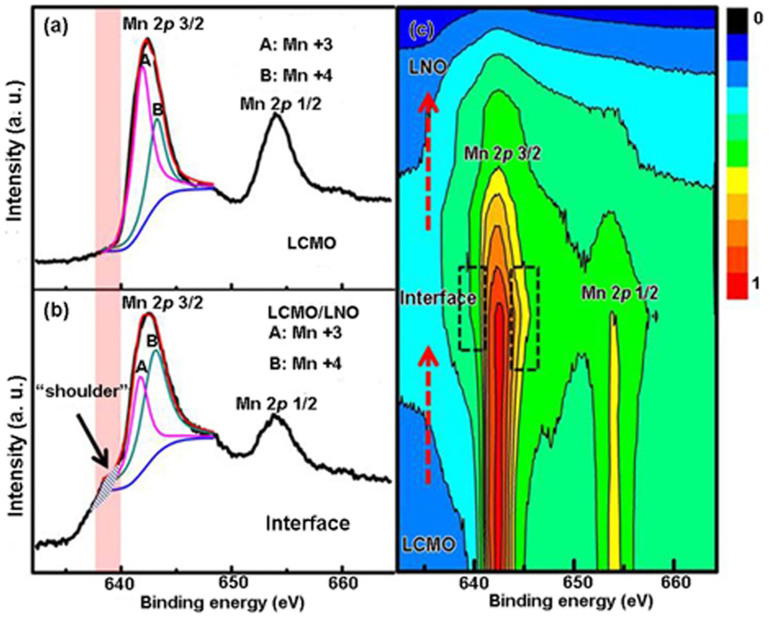
(a) Mn 2*p*-spectra of the Bulk LCMO that record about 4 nm to the LCMO/LNO interface. (b) Mn 2*p*-spectra at the LCMO/LNO interface. (c) A two-dimensional contour map of the Mn 2*p*_3/2_ and Mn 2*p*_1/2_ spectra, recorded all the data across the LCMO/LNO interface in the interface region.

Figure 4 shows the detailed core-level spectra of Mn 2*p* to analyze the valence states of Mn cations. It is found that the binding energies of Mn 2*p*_3/2_ and Mn 2*p*_1/2_ of the LCMO layer are 641.7 and 643.7 eV, respectively, as shown in figure 4(a). The Mn 2*p*_3/2_ spectrum can be fitted very well with two peaks at energies of 641.6 eV (noted as A) and 642.6 (noted as B) which belongs to Mn ions in 3+ and 4+ valence states, respectively. To ensure the quality of the peak fitting, all the core-level spectra were fitted with Gaussian-Lorentzian peaks and a combined Shirley background^39^. The Mn^3+^/Mn^4+^ ratio in the LCMO can be deduced by the two fitted peaks for the Mn 2*p*_3/2_ peak^40^. The result shows that Mn^3+^ is absolutely dominant in the LCMO layer, but there are small amount of Mn^4+^. Figure 4(b) shows Mn 2*p*-spectra at the LCMO/LNO interface. Compared with figure 4(a), it is clear that there are two pronounced features in the Mn 2*p*_3/2_ spectrum. Firstly, it is noted that there is a larger contribution of Mn^4+^ in the spectrum, indicating that the Mn^3+^/Mn^4+^ ratio decreased at the interface (Supplementary Fig. S3). Secondly, a pronounced “shoulder” structure with the energy feature at about 2 eV below the main line (marked by a blue shadow area and noted as “shoulder”) has been observed. The intensity of the shoulder structure systematically increases with increasing probing depth until the LCMO/LNO interface. Figure 4(c) displays the two-dimensional contour map of the Mn 2*p*_3/2_ and Mn 2*p*_1/2_ spectra, recorded all the data along the depth-resolved area, as shown in figure 3. By normalizing the Mn 2*p*_3/2_ peak in figure 4(a), these two features can be clearly observed in the interface region compared with the LCMO layer, as shown in the black dotted boxes in figure 4(c). According to the experimental results, on the one hand, charge transfer may happen in the interface region where the *e_g_* electrons of Mn^3+^ ion near *E_F_* may hop to nearby new states, decreasing the Mn^3+^/Mn^4+^ ratio. An average 0.22*e* (where *e* is the charge of the electron) per Mn ions can be roughly estimated for the charge transfer amplitude at the LCMO interface region. On the other hand, this kind of shoulder structure has been depicted as “well-screened” feature related to the doping-induced density of state of the *e_g_* valence electrons^41^,^42^. In particular, the screening feature is related to a nonlocal screening as reported by Veenendaal *et al*. in LSMO pure films^43^. Horiba *et al*. have demonstrated that the screening feature is from the new states at the Fermi level by using multi-cluster model calculation^41^. The experimental results suggest that there may be charge transfer and new states at the interface.

**Figure 5:**
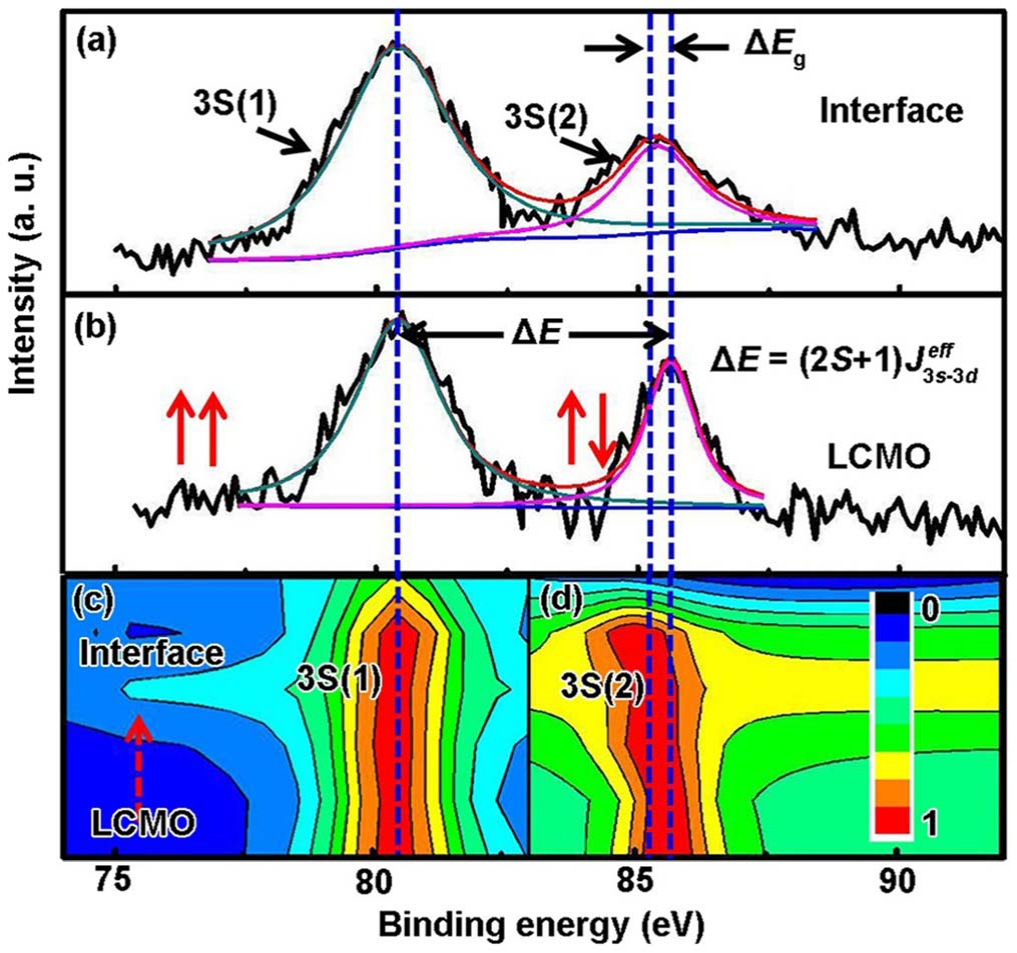
(a) and (b) Mn 3*s* core-level XPS spectra of the LCMO layer and the LCMO/LNO interface. (c) A two-dimensional contour map of the Mn 3*s* spectra, recorded all the fitting data from the LCMO to the LCMO/LNO interface.

To determine the valence change of the Mn ions at the interface, we also examined the core-level spectrum of Mn 3*s*. A Mn 3s spectrum is a definite indicator and quantitative determination for the Mn valence^44^,^45^,^46^ due to the fact that it is not interfered by other peaks thus the accuracy can be guaranteed. Figure 5(a) shows different splitting magnitudes in the binding energy of Mn 3*s* of the LCMO layer and the interface. The energy separation (*ΔE*) between the splitting peaks is derived from the different valence states of Mn ions due to the interaction between the 3*s* core hole and 3*d* electrons for the 3*d* transition metals^47^,^48^, and can be described by the following formula: *ΔE* = (2*S* + 1) *J*_3*s*−3*d*_; where *S* is the total spin moment and *J*_3*s*−3*d*_ is the effective exchange integral between Mn 3*d* and Mn 3*s* states^42^. A direct experimental result shows that the energy separation between the splitting peaks of a high-spin state at the lower binding energy noted as 3*S*(1) and a low-spin state at the higher binding energy noted as 3*S*(2). The values of the 3*s* level splitting is about 5.5 eV for Mn^3+^ and 4.5 eV for Mn^4+^ reported by Wu *et al.*^48^ There, the Mn valences can be derived from the 3s energy splitting *ΔE*. Figure 5(c) displays the two-dimensional contour map of the Mn 3*s* spectra, recorded all the fitting data along the depth-resolved area (supplementary Fig. S4). The data demonstrate a clear decrease of the Mn 3*s* exchange splitting from *ΔE* ~ 5.2 ± 0.1 eV for LCMO layer to *ΔE* ~ 4.9 ± 0.1 eV for the interface. Within the error bars we find an average 0.24*e* per Mn ions can be roughly estimated for the charge transfer amplitude at the LCMO layer of about 2 nm thicknesses, corresponding with the Mn 2*p* data.

**Figure 6:**
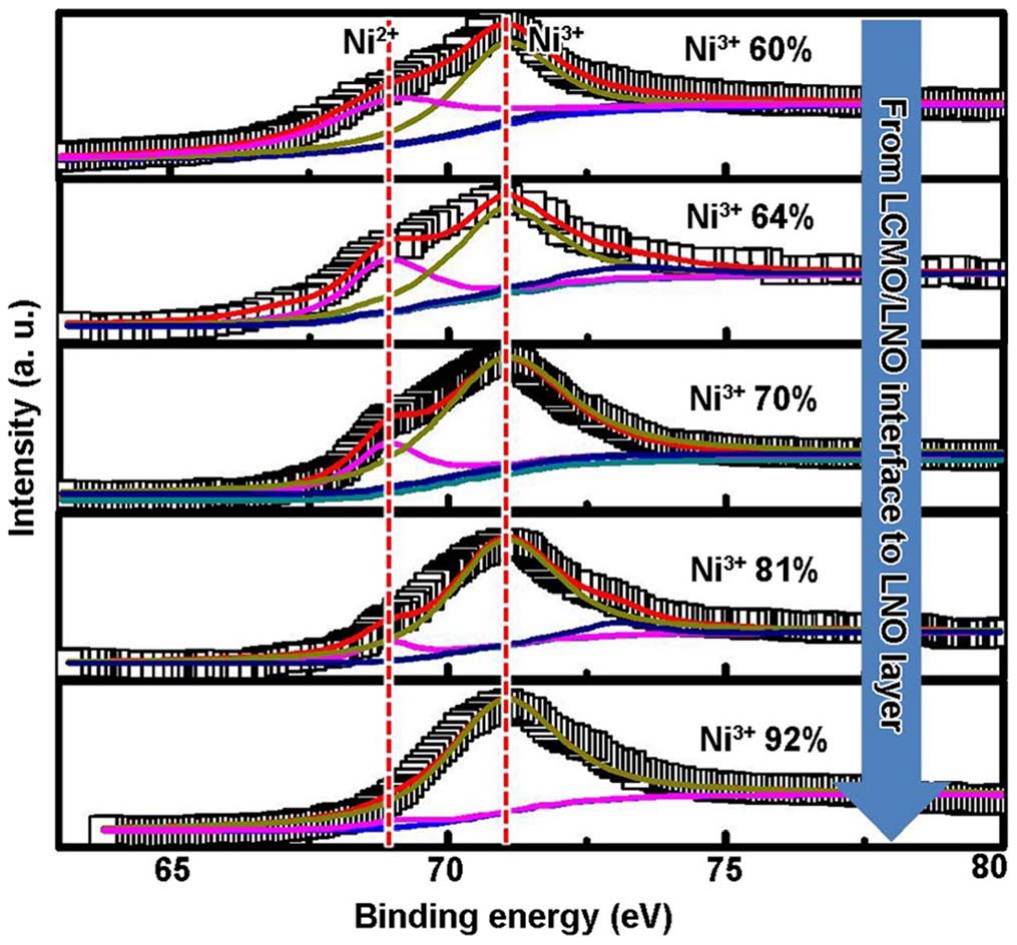
Ni 3*p* core-level XPS spectra from the LCMO/LNO interface to the LNO layer.

To further understand the exact charge distribution across the LCMO/LNO interface, it is possible to monitor the binding energies for the core-level electrons of Ni 2*p* and Ni 3*p* at the interface. The core-level spectra of Ni 2*p* and 3*p* of the LNO layer and the interface are compared in Supplementary Fig. S5. For comparison, the Ni 2*p* and 3*p* spectra of NiO were also measured. Usually, the La 3*d*_3/2_ peak partially overlaps with Ni 2*p*_3/2_ peak due to a small energy separation between La 3*d* and Ni 2*p* peaks. Therefore, it is difficult to distinguish the Ni^3+^ and Ni^2+^ in LNO. In this study, the intensity of the La 3*d*_3/2_ and Ni 2*p*_3/2_ peaks at the interface is larger than that of the LNO layer. Compared with the spectra of NiO, the larger intensity of this peak may be due to the part contribution of the Ni^2+^ because the 2*p*_3/2_ peak of Ni^2+^ has the same binding energy as the La 3*d*_3/2_ peak, while the 2*p*_3/2_ peak of Ni^3+^ is located at the higher binding energy. In order to discuss the precise valence of Ni at the LCMO/LNO interface, based on the method proposed by Qiao *et al.*^49^, it is convenient to use the Ni 3*p* spectra to fixed the Ni valence, as shown in figure 6. Figure 6 shows that the peaks at 69.0 eV (noted as B) and 70.7 eV (noted as C) are assigned to Ni^2+^ 3*p*_1/2_ and Ni^3+^ 3*p*_3/2_. For the LNO layer, the peak C for the Ni^3+^ 3*p*_3/2_ is absolutely dominant. In contrast, the peak B for the Ni^2+^ 3*p*_1/2_ significantly increases at the interface. This is in accordant with the experimental results above in the core-level spectra of Ni 2*p*. The Ni valence states can be described by the relative intensity ratio (RIR) of Ni 3*p*_3/2_/3*p*_3/2_ for Ni^2+^ and Ni^3+^. Spatially averaged XPS of Ni 3*p* has demonstrated that an average charge of 0.2*e* per Ni is transferred from Mn to Ni ions. The thickness with pronounced Ni^2+^ is about 1.6 nm in the LNO layer at the interface region.

**Figure 7:**
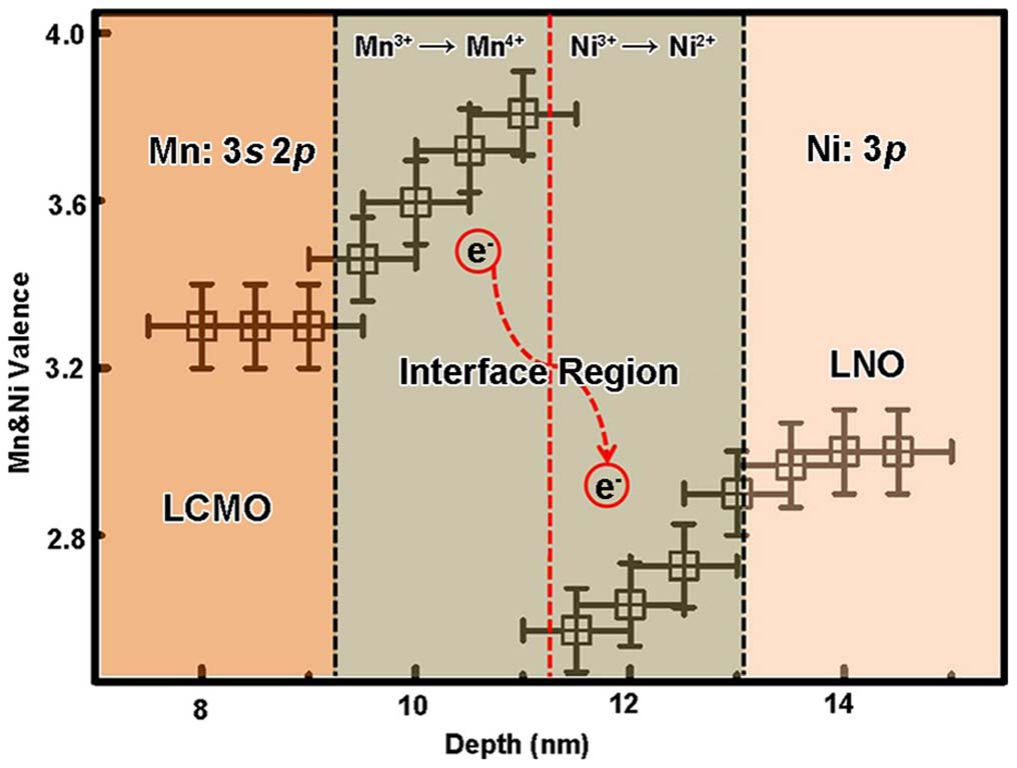
Mn and Ni valences of LCMO and LNO in the interface region.

**Figure 8:**
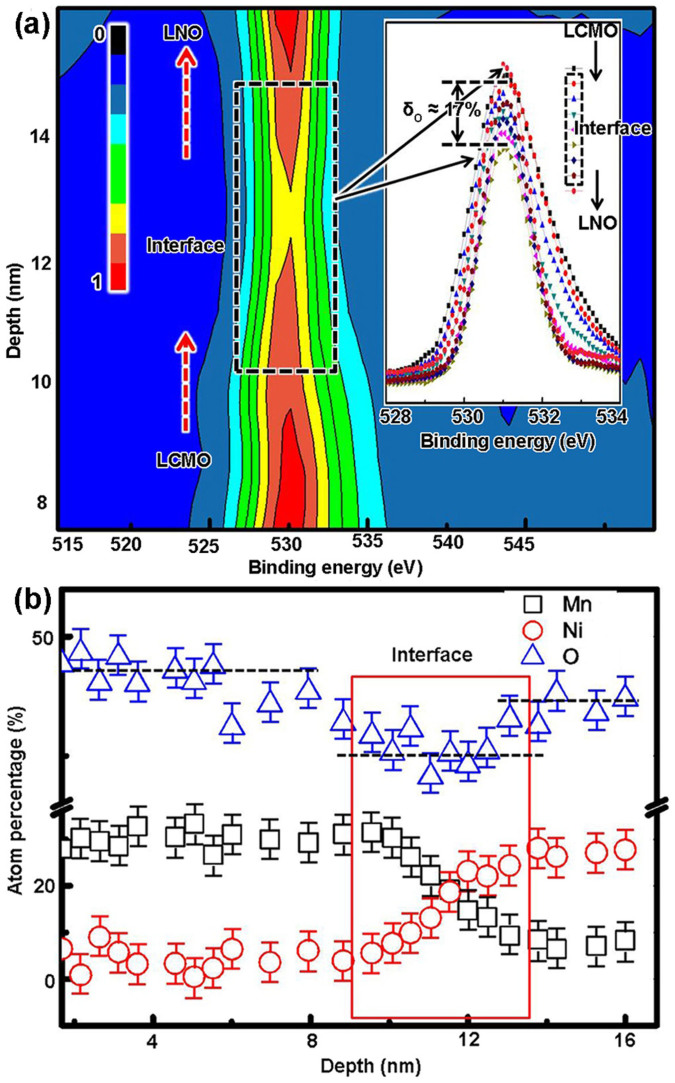
(a) A two-dimensional contour map of the O 1*s* spectra across the interface. Insert: O 1*s* spectra across the interface. (b) The detail information of atom percentages of the Mn, Ni and O across the LCMO/LNO interface.

Figure 7 shows the Mn and Ni valence change across the interface region. The presence of Mn^4+^ and Ni^2+^ ions in the interface region indicates that the charge disproportionation of the type: Mn^3+^ − Ni^3+^ → Mn^4+^ − Ni^2+^ occurs at the interface. The average ~0.2*e* charge transferred from Mn to Ni ions and occurred within about 4 nm thickness in the interface region. This site to site charge distribution at the LCMO/LNO interface is similar to the LNO/SrMnO_3_ (SMO) systems reported by Hoffman *et al*.^26^ As other oxides as proposed in the SrTiO_3_/LaAlO_3_ interface^10^,^12^,^13^,^14^, LCMO and LNO are oxygen-deficient materials, thus fluctuating valence in these materials is directly related to oxygen stoichiometry which will be adjusted to ensure charge neutrality and oxygen stoichiometry^22^,^50^. Interestingly, as shown in figure 8(a) and (b) (Supplementary Fig. S2), the oxygen composition decreased (δ_O_ ≈ 17%) in the interface region about 4 nm thickness, corresponding with the thickness of the Mn and Ni charge transfer region. This result reveals that the charge transfer between Mn and Ni ions occurs with the oxygen vacancies in the interface region. It is noted that a new ground state could form as the charge redistribution between the Mn and Ni ions in the interface region which will determine the physical properties such as FM interaction at the interfaces in the LSMO/YBCO and LNO/LMO systems^17^,^27^.

## Discussion

**Figure 9:**
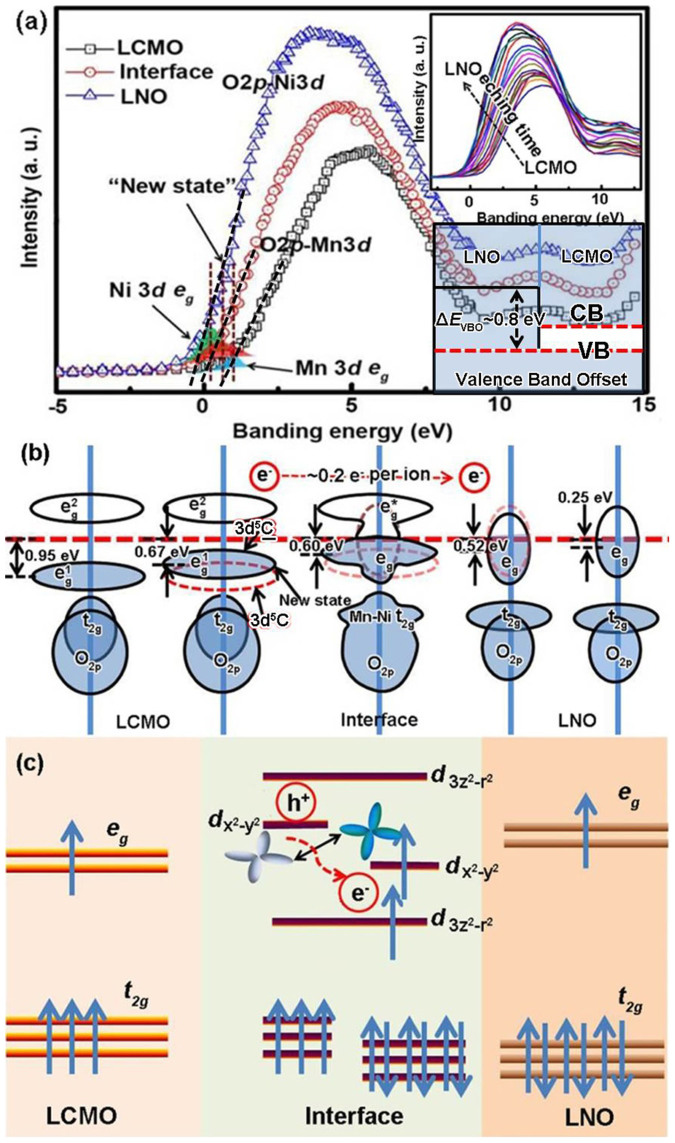
(a) Survey scans of the valence band record in the LCMO and LNO layers, and at the LCMO/LNO interface. The shadow areas highlight the spectral regions of the *e_g_* close to *E_F_*. Insert: the valence band continuously shifts from the LCMO layer to the LNO layer and the valence band offset at the LCMO/LNO interface. (b) A schematic of the density of states and the *e_g_* band transitions across the interface. (c) A schematic energy diagram showing the orbital reconstruction at the LCMO/LNO interface.

Having established this, we now explore the physical origin of the charge transfer between Mn and Ni 3*d*-ions at the interface. In general, charge transfer is determined by the prerequisite of the energy continuity to unify the Fermi level at an interface. Under this circumstance, direct observation of the valence band at the LCMO/LNO interface can reveal the origin of the charge transfer. Figure 9(a) shows the 2*p*-3*d* spectra of the LCMO and LNO layers, and the LCMO/LNO interface. There is a large gap between the Fermi levels of the LCMO and LNO, and the Fermi level of the interface is between both sides. The valence band offset (VBO) at the LCMO/LNO interface can be determined to be *ΔE*_VBO_ ~ 0.8 eV (Supplementary Fig. S6) by using a linear regression fit (dashed black line in figure 9(a)) along the leading edge of the valence band of LCMO and LNO. Thus, the valence band of LCMO is shifted to higher binding energy compared to LNO. The upper insert in figure 9(a) shows that the Fermi level continuously shifts from the LCMO layer to the LNO layer in the interface region. It is noted that, as described in the conventional band structure of the semiconductor heterointerfaces^51^, the prerequisite of the energy continuity at the LCMO/LNO interface brings about the energy alignment. The shifting of the Fermi level will modify and flatten the barrier height of the electron orbitals at the interface. In analogy to atoms in a free space, the electrons thus have the choice of several types of energetically nearly equivalent electronic orbitals. Thus, the charge transfer happened as the Fermi level continuously shifts at the LCMO/LNO interface. The density of states (DOS) near the Fermi level are associate with the Mn and Ni 3*d*-*e_g_* electrons of LCMO and LNO. The Mn and Ni *e_g_* electrons can be presented by the blue and green shadow areas according to the LCMO and LNO, respectively, as reported in Refs. 52 and 53. The center of the *e_g_* band is at about 0.95 eV and 0.25 eV for the LCMO and LNO layers (Fermi level edge was fixed at 0 eV), respectively. Figure 9(b) shows a sketch of the LCMO, LNO and the interface density of states (DOS) based on the valence band structure. An average of about 0.1 eV per nearest-neighbor units of LCMO and LNO *e_g_* band center has been shown. Given the observation, the electron's hopping may occurs from an occupied state to an unoccupied state of nearly the same energy in the nearest neighbor LCMO units. The procedure can be described as an electron hopping from one *e_g_* band 3*d* ^5^C (note as dashed red line) to another *e_g_* band 3*d* ^5^C, as shown in figure 8(b), where C represents doping-induced states at *E_F_* and 3*d* ^5^C results from a charge transfer from C to the Mn 3*d* state. Compared with the nearest neighbor *e_g_* band, the new *e_g_* band can be described as a new state. Therefore, the valence band at the LCMO/LNO interface can be described as a “new state” at *E_F_*. The “new state” can also be described as the doping-induced *e_g_* bandwidths shift in the LCMO near the interface. It is interesting to bring these experimental results and the shoulder feature in the Mn 2*p* spectra together to explain the well-screened feature of the LCMO near the interface. The *e_g_* electron become more itinerant with the depth to the LCMO/LNO interface, thus the shoulder feature become more pronounced.

In this context, we now turn to explore the origin of FM interaction at the LCMO/LNO interface as well as the resulting exchange bias coupling. Based on the experimental results of the Fermi level shifting and charge transfer mode of the 3*d* ions, the schematic energy diagram of the orbital reconstruction at the LCMO/LNO interface can be shown in figure 9(c). It should be noted that the energy level of the 3*d*-*t_2g_* of the Mn and Ni will not change due to the fact that the Ni *t_2g_* is in the PM state and the coupling strength between the 3*d*-*t_2g_* of Mn and Ni is small. Under this circumstance, the energy level of the 3*d*-*t_2g_* orbitals of Mn and Ni are substantially the same as the inner layer of the LCMO and LNO, as shown in figure 8(b). As mentioned above, as the charge transfer from Mn to Ni ions, the covalent bond of Mn-O-Ni forms at the interface. The strong hybridization between the Mn and Ni orbitals forms the bonding orbital *d*3*z*^2^-*r*^2^ (lower energy) and the corresponding antibonding orbital *d*3*z*^2^-*r*^2^ (higher energy) at the interface. The charge hopping process should be via the apical oxygen ion at the interface. As the charge transfer happened, these new electron orbitals are occupied by *e_g_*-electrons with equivalent (or degenerate) energy. Hence, the bonding orbital *d*3*z*^2^-*r*^2^ is occupied by an electron. At the same time, the corresponding oxygen *p* hole formed at the same *e_g_* symmetry of the Ni *e_g_* states. The oxygen hole coupled with the Mn 3*d*-*e_g_* electrons due to the same *e_g_* symmetry and a similar energy level at the interface. Namely, the antibonding orbital *d*3*z*^2^-*r*^2^ is occupied by the hole hopped from the oxygen ions. The *dx*^2^-*y*^2^ orbital is also occupied by an electron as the *c*-axis compression of NiO_6_ octahedra imposed by the epitaxial stain due to the STO substrate with a larger lattice parameter than that of the LNO layer^54^. The charge transfer across the interface leads to rearrangement of the Ni *e_g_* orbital, so that the Ni *d*x^2^-y^2^ orbital is occupied by the electron hopped from the Mn *d*x^2^-y^2^ orbital. The *e_g_* electron of the Mn^3+^ hops to the Ni *dx*^2^-*y*^2^ orbital, favoring the energy stable. The filling of the orbital would affect the interfacial magnetic couple effect when the *e_g_* electrons are associated with the double-exchange interaction model^55^, such as the experiment result of the unexpected exchange bias in the LNO/LaMnO_3_ (LMO) bilayer^17^. The alternating occupation of *dx*^2^-*y*^2^ orbitals of the Ni and Mn on neighbor lattice sites favors ferromagnetism. Thus double exchange interaction formed at the interface through Mn^4+^-O-Ni^2+^, as shown in figure 8(b). The FM order at the interface is determined by the interplay between Mn 3*d*-*t_2g_* and *e_g_* electrons. The FM interaction between Ni^2+^ and Mn^4+^ gives rise to magnetic regions that pin the FM LCMO and cause exchange bias coupling at the LCMO/LNO interface.

## Methods

LCMO(25 nm)/LNO(35 nm) bilayers were grown on STO (001) single-crystal substrates (with a cubic structure and the lattice parameter a = 0.391 nm) by pulsed laser deposition (PLD) using a KrF (λ = 248 nm) excimer laser. For comparison, pure single LCMO, LNO and NiO layers were also grown on STO substrates under the same conditions. Structural quality and lattice parameters of the samples were analyzed by X-ray diffraction (XRD) (Rigaku, D/max-2000, CuKα radiation). Surface morphology was characterized by atomic force microscopy (AFM). Microstructure and thickness of the films were observed by transmission electron microscopy (TEM) (F20, Tecnai). Epitaxy between the interfaces of LCMO/LNO and LNO/STO was also confirmed by high-resolution TEM (HRTEM). The chemical states of the ions in the LCMO/LNO interface region were determined by X-ray photoelectron spectroscopy (XPS), (Therma ESCALAB 250; Al Kα source, 1486.60 eV, Resolution: 400 meV, Energy step: 0.1 eV). Survey spectra and the following core levels were studied: Mn 2*p*, Mn 3*s*, Ni 2*p*, Ni 3*p*, La3*d*. Magnetization measurements were performed from 5 to 300 K and external magnetic fields up to 3 k*Oe* using a superconducting quantum interference device magnetometer (SQUID, Quantum Design).

## Author Contributions

Z.W. and Z.Z. conceived and designed the experiments. X.N. and Z.W. carried out the experiments and fulfilled data analysis. X.N. and Z.W. wrote the paper. X.N., Z.W. and Z.Z. discussed the results and commented on the manuscript.

## Supplementary Material

Supplementary Information Supplementary information

## References

[b1] Holden T. *et al.* Proximity induced metal-insulator transition in YBa_2_Cu_3_O_7_/La_2/3_Ca_1/3_MnO_3_ superlattices. Phys. Rev. B 69, 064505 (2004).

[b2] Dybko K., Aleshkevych P., Sawicki M., Paszkowicz W. & Przyslupski P. The onset of ferromagnetism and superconductivity in [La_0.7_Sr_0.3_MnO_3_(*n* u. c.)/YBa_2_Cu_3_O_7_(2 u. c.)]_20_. J. Phys.: Condens. Matter. 25, 376001 (2013). 23962975 10.1088/0953-8984/25/37/376001

[b3] Ding J. F., Tian Y. F., Hu W. J., Lin W. N. & Wu T. Exchange coupling and coercivity enhancement in cuprate/manganite bilayers. Appl. Phys. Lett. 102, 032401 (2013).

[b4] Ning X. K., Wang Z. J. & Zhang Z. D. Exchange bias effect and large coercivity enhancement in SrRuO_3_/NiO multilayer. J. Phys. D: Appl. Phys. 46, 452001 (2013).

[b5] Reyren N. *et al.* Superconducting interfaces between insulating oxides. Science 317, 1196 (2007). 17673621 10.1126/science.1146006

[b6] Dikin D. A. *et al.* Coexistence of superconductivity and ferromagnetism in two dimensions. Phys. Rev. Lett. 107, 056802 (2011). 21867087 10.1103/PhysRevLett.107.056802

[b7] Yang K. Y. *et al.* Possible interaction-driven topological phase in (111) bilayers of LaNiO_3_. Phys. Rev. B 84, 201104(R) (2011).

[b8] Li L., Richter C., Mannhart J. & Ashoori R. C. Coexistence of magnetic order and two-dimensional superconductivity at LaAlO_3_/SrTiO_3_ interface. Nat. Phys. 7, 762 (2011).

[b9] Bert J. A. *et al.* Direct imaging of the coexistence of ferromagnetism and superconductivity at the LaAlO_3_/SrTiO_3_ interface. Nat. Phys. 7, 767 (2011).

[b10] Kalabukhov A. *et al.* Effect of oxygen vacancies in the SrTiO_3_ substrate on the electrical properties of the LaAlO_3_/SrTiO_3_ interface. Phys. Rev. B 75, 121404(R) (2007).

[b11] Crumlin E. J. *et al.* In situ ambient pressure x-ray photoelectron spectroscopy of cobalt perovskite surfaces under cathodic polarization at high temperatures. J. Chem. Phys. C 117, 16087 (2013).

[b12] Herranz G. *et al.* High Mobility in LaAlO_3_/SrTiO_3_ Heterostructures: origin, dimensionality, and perspectives. Phys. Rev. Lett. 98, 216803 (2007). 17677799 10.1103/PhysRevLett.98.216803

[b13] Siemons W. *et al.* origin of charge density at LaAlO3 on SrTiO3 heterointerfaces: possibility of intrinsic doping. Phys. Rev. Lett. 98, 196802 (2007). 17677645 10.1103/PhysRevLett.98.196802

[b14] Eckstein J. N. Watch out for the lack of oxygen. Nature Mater. 6, 473–474 (2007). 17603525 10.1038/nmat1944

[b15] Salluzzo M. *et al.* Orbital reconstruction and the two-dimensional electron gas at the LaAlO_3_/SrTiO_3_ interface. Phys. Rev. Lett. 102, 166804 (2009). 19518739 10.1103/PhysRevLett.102.166804

[b16] Grutter A. J. *et al.* Interfacial ferromagnetism in LaNiO_3_/CaMnO_3_ superlattices. Phys. Rev. Lett. 111, 087202 (2013). 24010469 10.1103/PhysRevLett.111.087202

[b17] Gilbert M., Zubko P., Scherwitzl R., Iniguez J. & Triscone J. M. Exchange bias in LaNiO_3_-LaMnO_3_ superlattices. Nature Mater. 11, 195–198 (2012). 22266467 10.1038/nmat3224

[b18] Yu P. *et al.* Interface ferromagnetism and orbital reconstruction in BiFeO_3_-La_0.7_Sr_0.3_MnO_3_ heterostructures. Phys. Rev. Lett. 105, 027201 (2010). 20867733 10.1103/PhysRevLett.105.027201

[b19] Ke X., Rzchowski M. S., Belenky L. J. & Eom C. B. Positive exchange bias in ferromagnetic La_0.67_Sr_0.33_MnO_3_/SrRuO_3_ bilayers. Appl. Phys. Lett. 84, 5458 (2004).

[b20] Rana R., Pandey P. & Rana D. S. Controlling the coexisting vertical magnetization shift and exchange bias in La_0.3_Sr_0.7_FeO_3_/SrRuO_3_ bilayers. Appl. Phys. Lett. 104, 092413 (2014).

[b21] Padhan P., Prellier W. & Budhani R. C. Antiferromagnetic coupling and enhanced magnetization in all-ferromagnetic superlattices. Appl. Phys. Lett. 88, 192509 (2006).

[b22] Joseph Joly V. L., Joy P. A., Date S. K. & Gopinath C. S. Two ferromagnetic phases with different spin states of Mn and Ni in LaMn_0.5_Ni_0.5_O_3_. Phys. Rev. B 65, 184416 (2002).

[b23] Tebano A. *et al.* Evidence of orbital reconstruction at interfaces in ultrathin La_0.67_Sr_0.33_MnO_3_ Films. Phys. Rev. Lett. 100, 137401 (2008). 18517994 10.1103/PhysRevLett.100.137401

[b24] Lee J.-S. *et al.* Controlling competing interactions at oxide interfaces: Enhanced anisotropy in La_0.7_Sr_0.3_MnO_3_ films via interface engineering. Phys. Rev. B 85, 235125 (2012).

[b25] Rojas Sánchez J. C., Nelson-Cheeseman B., Granada M., Arenholz E. & Steren L. B. Exchange-bias effect at La_0.75_Sr_0.25_MnO_3_/LaNiO_3_ interfaces. Phys. Rev. B 85, 094427 (2012).

[b26] Hoffman J. *et al.* Charge transfer and interfacial magnetism in (LaNiO_3_)*_n_*/(LaMnO_3_)_2_ superlattices. Phys. Rev. B 88, 144411 (2013).

[b27] Przyslupski P. *et al.* Magnetic properties of La_0.67_Sr_0.33_MnO_3_/YBa_2_Cu_3_O_7_ supperlattices. Phys. Rev. B 69, 134428 (2004).

[b28] Yamada H. *et al.* Engineered interface of magnetic oxides. Science 305, 646 (2004). 15286367 10.1126/science.1098867

[b29] Zhou W. *et al.* Charge transfer and Fermi level shift in *p*-doped sigle-walled carbon nanotubes. Phys. Rev. B 71, 205423 (2005).

[b30] Osada M. *et al.* Orbital reconstruction and interface ferromagnetism in self-assembled nanosheets superlattices. ACS Nano. 5, 6871 (2011). 21823647 10.1021/nn200835v

[b31] Klein A. Energy band alignment at interfaces of semiconducting oxides: A review of experimental determination using photoelectron spectroscopy and comparison with theoretical predictions by the electron affinity rule, charge neutrality levels, and the common anion rule. Thin Solid Film. 520, 3271 (2012).

[b32] Greiner M. T., Helander M. G., Wang Z. B., Tang W. M. & Lu Z. H. Effects of Processing Conditions on the Work Function and Energy-Level Alignment of NiO Thin Films. J. Phys. Chem. C 114, 19777 (2010).

[b33] Moutis N., Christides C., Panagiotopoulos I. & Niarchos D. Exchange-coupling properties of La_1-x_Ca_x_MnO_3_ ferromagnetic/antiferromagnetic multilayers. Phys. Rev. B 64, 094429 (2001).

[b34] Ding J. F. *et al.* Interfacial spin glass state and exchange bias in manganite bilayers with competing magnetic orders. Phys. Rev. B 87, 054428 (2013).

[b35] Ning X. K., Wang Z. J., Zhao X. G., Shih C. W. & Zhang Z. D. Exchange bias in La_0.7_Sr_0.3_MnO_3_/NiO and LaMnO_3_/NiO interfaces. J. Appl. Phys. 113, 223903 (2013).

[b36] Ning X. K., Wang Z. J. & Zhang Z. D. Large, temperature-tunable low-field magnetoresistance in La0.7Sr0.3MnO3:NiO nanocomposite films modulated by microstructures. Adv. Funct. Mater. 24, 5393 (2014).

[b37] Cai J. W., Liu K. & Chien C. L. Exchange coupling in the paramagnetic state. Phys. Rev. B 60, 72 (1999).

[b38] Gao G. Y., Jin S. W. & Wu W. B. Lattice-mismatch-strain induced inhomogeneities in epitaxial La_0.7_Ca_0.3_MnO_3_ films. Appl. Phys. Lett. 90, 012509 (2007).

[b39] Shirley D. High-resolution X-ray photoemission spectrum of the valence band of gold. Phys. Rev. B 5, 4709 (1972).

[b40] Morilla-Santos C., Schreiner W. H. & Lisboa-Filho P. N. Chemical deposition of La_0.7_Ca_0.3_MnO_3 ± δ_ films on ceramic substrates. Mat. Res. 14, 217 (2011).

[b41] Horiba K. *et al.* Nature of the well screened state in hard x-ray Mn 2*p* core-level photoemission measurement of La_1-x_Sr_x_MnO_3_ films. Phys. Rev. Lett. 93, 236401 (2004). 15601180 10.1103/PhysRevLett.93.236401

[b42] Schlueter C. *et al.* Evidence of electronic band redistribution in La0.65Sr0.35MnO3-δ by hard x-ray photoelectron spectroscopy. Phys. Rev. B 86, 155102 (2012).

[b43] Van Veenendaal M. Competition between screening channels in core-level x-ray photoemission as a probe of changes in the ground-state properties of transition-metal compounds. Phys. Rev. B 74, 085118 (2006).

[b44] Zhao L. Z. & Young V. XPS studies of carbon supporten films formed by the resistive deposition of manganese. J. Electron Spectrosc. Relat. Phenom. 34, 45 (1984).

[b45] Galakhov V. R. *et al.* Mn 3*s* exchange splitting in mixed-valence manganites. Phys. Rev. B 65, 113102 (2002).

[b46] Beyreuther E., Grafström S., Eng L. M., Thiele C. & Dörr K. XPS investigation of Mn valence in lanthanum manganite thin films under variation of oxygen content. Phys. Rev. B 73, 155425 (2006).

[b47] Brabers V. A. M., Van Setten F. M. & Knapen P. S. A. X-ray photoelectron spectroscopy study of the cation valences in nickel manganites. J. Solid State Chem. 49, 93 (1983).

[b48] Wu Q. H., Liu M. L. & Jaegermann W. X-ray photoelectron spectroscopy of La_0.5_Sr_0.5_MnO_3_. Mater. Lett. 59, 1980 (2005).

[b49] Qiao L. & Bi X. F. Direct observation of Ni^3+^ and Ni^2+^ in correlated LaNiO_3-δ_ films. Europhys. Lett. 93, 57002 (2011).

[b50] Guo H. Z. *et al.* Influence of defects on structural and magnetic properties of multifunctional La_2_NiMnO_6_ thin films. Phys. Rev. B 77, 174423 (2008).

[b51] Ohtomo A., Muller D. A., Grazul J. L. & Hwang H. Y. Artificial charge-modulation in atomic-scale perovskite titanate superlattices. Nature 419, 378–380 (2002). 12353030 10.1038/nature00977

[b52] Park J.–H. *et al.* Electronic aspects of the ferromagnetic transition in manganese perovskites. Phys. Rev. Lett. 76, 4215 (1996). 10061230 10.1103/PhysRevLett.76.4215

[b53] Gray A. X. *et al.* Insulating state of ultrathin epitaxial LaNiO3 thin films detected by hard x-ray photoemission. Phys. Rev. B 84, 075104 (2011).

[b54] Chaloupka J. & Khaliullin G. Orbital order and possible superconductivity in LaNiO_3_/LaMnO_3_ superlattices. Phys. Rev. Lett. 100, 016404 (2008). 18232795 10.1103/PhysRevLett.100.016404

[b55] Zener C. *et al.* Interaction between the d-shells in the transition metals. II. Ferromagnetic compounds of manganese with perovskite structure. Phys. Rev. 82, 403 (1951).

