# Supplementary material for: Fermi Level shifting, Charge Transfer and Induced Magnetic Coupling at La0.7Ca0.3MnO3/LaNiO3 Interface
Source: Sci Rep. 2015 Feb 13;5:8460. doi: 10.1038/srep08460 (PMC4327574; doi:10.1038/srep08460)
Supplement: Supplementary Information [file srep08460-s1.pdf]

Supplementary information

Fermi Level shifting, Charge Transfer and Induced Magnetic Coupling at $La_{0.7}Ca_{0.3}MnO_3/LaNiO_3$ Interface

Xingkun Ning, Zhanjie Wang, and Zhidong Zhang*

Shenyang National Laboratory for Materials Science, Institute of Metal Research (IMR), Chinese

Academy of Sciences (CAS), 72 Wenhua Road, Shenyang 110016, P. R. China.

E-mail:wangzj@imr.ac.cn

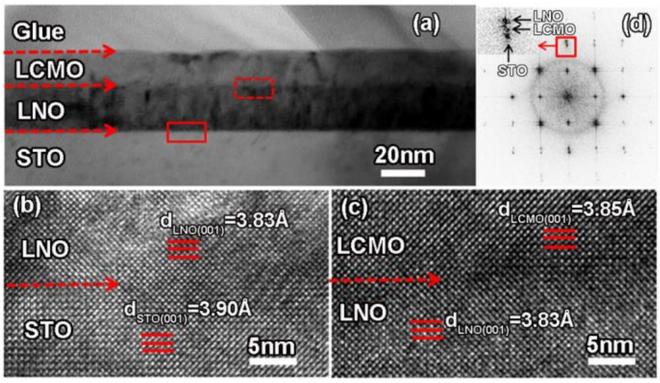

Figure S1. A low-magnification TEM micrograph of the LCMO/LNO bilayer with thickness of 25 nm and 35 nm for the LCMO and LNO layers, respectively; (b-c) High-resolution TEM (HRTEM) images of the STO/LNO and LNO/LCMO interfaces; (d) Fast Fourier Transformation (FFT) patterns transformed from the HRTEM images of (b) and (c).

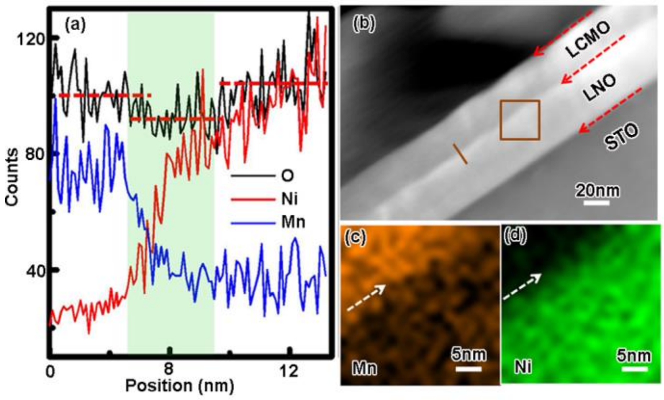

Figure S2. (a) Plots of O, Ni and Mn contents across the LCMO/LNO interface. (b) A low-magnification HAAD STEM image of the LCMO/LNO sample and the elemental mappings of (c) Mn and (d) Ni.

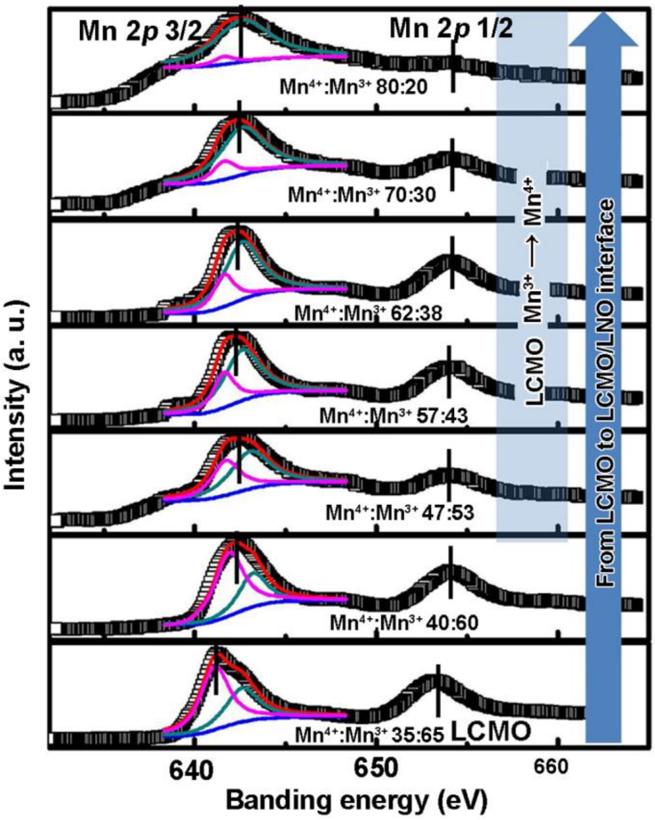

Figure S3. Mn 2p-spectra from the LCMO layer to the LCMO/LNO interface.

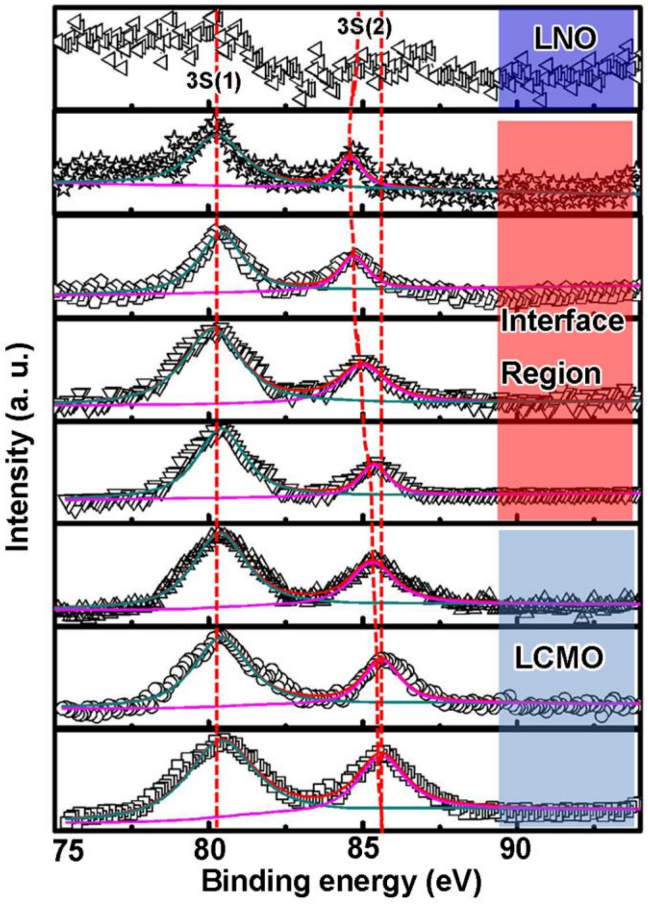

Figure S4. Mn 3*s* core-level XPS spectra from LCMO layer to the LCMO/LNO interface.

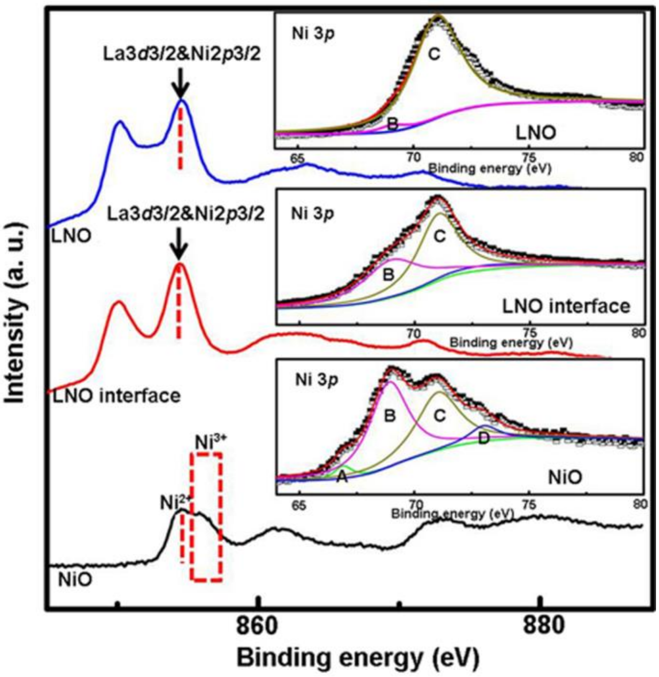

Figure S5. Ni 2*p* core-level XPS spectra of pure NiO, the LCMO/LNO interface and the LNO layer. Insert: the corresponding Ni 3*p* core-level XPS spectra of pure NiO, the LCMO/LNO interface and the LNO layer.

Figure S5 shows that the intensity of the La 3$d_{3/2}$ and Ni 2$p_{3/2}$ peaks at the interface is larger than that of the LNO layer. Compared with the spectra of NiO, the larger intensity of this peak may be due to the part contribution of the Ni^{2+} because the 2$p_{3/2}$ peak of Ni^{2+} has the same binding energy as the La 3$d_{3/2}$ peak, while the 2$p_{3/2}$ peak of Ni^{3+} is located at the higher binding energy. Inserts of figure. S5 present the core-level spectra of Ni 3*p* of NiO, the LNO layer and the interface. The spin-orbit splitting energy between Ni 3$p_{3/2}$ and Ni 3$p_{1/2}$ peaks is taken as 2 eV. The peaks at 67.0 eV (noted as A) and 70.7 eV (noted as C) are assigned to Ni^{2+} 3$p_{3/2}$ and Ni^{3+} 3$p_{3/2}$, while the corresponding peaks of Ni^{2+} 3$p_{1/2}$ and Ni^{3+} 3$p_{1/2}$ are noted as B and D, respectively. For NiO, both charge states (2+ and 3+) are simultaneously presence but 2+ state appears to be dominant (peak B). For the LNO layer, the Ni 3*p* peak could be fitted by only two peaks, and the peak C for the Ni^{3+} 3$p_{3/2}$ is absolutely dominant. In contrast, the peak B for the Ni^{2+} 3$p_{1/2}$ significantly increases at the interface. This is in accordant with the experimental results above in the core-level spectra of Ni 2*p*. The thickness of the interface region with pronounced Ni^{2+} is about 2 nm in the LNO layer.

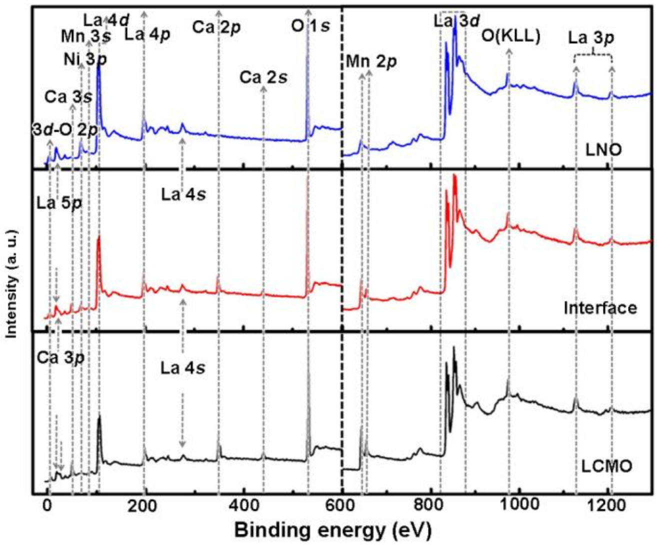

Figure S6. XPS survey spectra of the LCMO layer, LNO layer and the LCMO/LNO interface.

The valence band offset (VBO) at the LCMO/LNO interface is obtained from the following equation [1]: $\Delta E_{VBO(LCMO/LNO)} = (E_{CL(LCMO\text{-}I)} - E_{CL(LCMO\text{-}I)}) - [(E_{CL(LCMO)} - E_{V(LCMO)}) - (E_{CL(LNO)} - E_{V(LNO)})]$, where $\Delta E_{VBO(LCMO/LNO)}$ is the VBO of the LCMO relative to LNO (if positive, the valence band of LCMO is shifted to higher binding energy compared to LNO, and if negtive, it is shifted to lower binding energy), $E_{CL(LCMO\text{-}I)}$ and $E_{CL(LCMO\text{-}I)}$ is the binding energy of a core level LCMO and LNO at the interface, $E_{V(LCMO)}$ and $E_{V(LNO)}$ is the valence-band maxima (VBM) for the LCMO and LNO iner layer. Ni 3*p* and Ca 2*p* core level are used due to the fact that these two atomic core level are unique to a single layer of the bilayer. It also noted that these two core levels are intense and narrow in energy width compared with other core levels such as Mn 3*s* and Ca 2*s*.

Reference:

[1] Chambers, S. A. *et al.* Band discontinuities at epitaxial $SrTiO_3$/Si(001) heterojunctions. *Appl. Phys. Lett.* **77**, 1662 (2000).
